# Supplementary material for: Geometric analysis enables biological insight from complex non-identifiable models using simple surrogates
Source: PLoS Comput Biol. 2023 Jan 20;19(1):e1010844. doi: 10.1371/journal.pcbi.1010844 (PMC9891533; doi:10.1371/journal.pcbi.1010844)
Supplement: S1 File — (PDF) [file pcbi.1010844.s001.pdf]

Supplementary material for "Geometric analysis enables biological insight from complex non-identifiable models using simple surrogates"

Alexander P Browning[1,2,3] and Matthew J Simpson[*1,2]

[1]*School of Mathematical Sciences, Queensland University of Technology, Brisbane, Australia*
[2]*QUT Centre for Data Science, QUT, Australia*
[3]*Mathematical Institute, University of Oxford, Oxford, United Kingdom*

January 6, 2023

Contents

1 Numerical solution to the Ward and King model **2**

2 Sensitivity matrix **4**

3 Sensitivity of the Greenspan to bounded Gompertz model-map **5**

4 Sensitivity of the Greenspan to Richards model-map **5**

[*]Corresponding author: matthew.simpson@qut.edu.au

1 Numerical solution to the Ward and King model

Here, we briefly detail our numerical algorithm to approximate the solution of the Ward and King model [1], described by the coupled system of partial differential equations

$$
\begin{aligned}
\frac{\partial n}{\partial t} + v\frac{\partial n}{\partial r} &= n\frac{D_p}{r^2}\frac{\partial}{\partial r}\left(r^2\frac{\partial n}{\partial r}\right) + (k_m(c) - k_d(c))n, && t > 0, \quad 0 < r < R(t), \\
0 &= \frac{1}{r^2}\frac{\partial}{\partial r}\left(r^2\frac{\partial c}{\partial r}\right) - k(c)n, && t > 0, \quad 0 < r < R(t), \\
0 &= \frac{D_p}{r^2}\frac{\partial}{\partial r}\left(r^2\frac{\partial n}{\partial r}\right) + \frac{1}{r^2}\frac{\partial (r^2 v)}{\partial r} && t > 0, \quad 0 < r < R(t), \\
n(r,t) &= n_0, \qquad r(t) = R_0, && t = 0, \quad 0 < r < R(t), \\
\frac{\partial n}{\partial r} &= 0, \qquad \frac{\partial c}{\partial r} = 0, \qquad v = 0, && t > 0, \quad r = 0, \\
c = 1, \quad -D_p\frac{\partial n}{\partial r} &= -Q_p(1 - p_0 - n), \qquad \frac{\mathrm{d}R(t)}{\mathrm{d}t} = v, && t > 0, \quad r = R(t).
\end{aligned}
\tag{1}
$$

Our algorithm is implemented in Julia and is available on GitHub at `Modules/SpheroidModels/models/spatial/wardandking.jl`.

First, we follow [1] and non-dimensionalise both spatial and temporal variables such that $r = R_0\hat{r}$, $R(t) = R_0\hat{R}(t)$ and $t = \hat{t}/\lambda$. Without loss of generality, we then solve the non-dimensionalised system and re-dimensionalise the result to compare with experimental data. To compute the solution to the non-dimensionalised system, we next transform the growing domain $0 < \hat{r} < \hat{R}(t)$ to the fixed domain $0 < \xi < 1$ such that $r = \xi R(t)$. On the interior of the domain, the non-dimensionalisation and transformation to the fixed domain yields

$$
\frac{\partial n}{\partial \hat{t}} = \frac{D_p n}{\xi^2 \hat{R}^2(t)}\frac{\partial}{\partial \xi}\left(\xi^2\frac{\partial n}{\partial \xi}\right) - \frac{\hat{v} - \xi\hat{R}'(t)}{\hat{R}(t)}\frac{\partial \hat{n}}{\partial \xi} + (\hat{k}_m(c) - \hat{k}_d(c))n, \tag{2}
$$

$$
0 = \frac{1}{\xi^2}\frac{\partial}{\partial \xi}\left(\xi^2\frac{\partial c}{\partial \xi}\right) - \hat{R}^2(t)\hat{k}(c)n, \tag{3}
$$

and

$$
\frac{1}{\xi^2}\frac{\partial(\xi^2\hat{v})}{\partial \xi} = -\frac{1}{\hat{R}(t)}\frac{D_p}{\xi^2}\frac{\partial}{\partial \xi}\left(\xi^2\frac{\partial n}{\partial \xi}\right). \tag{4}
$$

Equation (4) can be integrated directly to yield

$$
\hat{v} = -\frac{D_p}{R(t)}\frac{\partial n}{\partial \xi}. \tag{5}
$$

We then solve eqs. (2) and (3) using a method-of-lines type approach, applying a spatial discretisation and expressing the resultant system of differential-algebraic-equations (DAEs) as

$$
M\frac{\mathrm{d}\mathbf{x}}{\mathrm{d}\hat{t}} = \mathbf{f}(\mathbf{x}), \tag{6}
$$

where $\mathbf{x}(t) = \left[R(t), n_1(t), n_2(t), ..., c_1(t), c_2(t), ...\right]^\intercal$, and M is a mass-matrix. Equation (6) is then solved using an Implicit-Euler scheme for DAEs implemented in `DifferentialEquations.jl` [2]. A Newton-Raphson algorithm is used to find an initial condition for $\left[c_1(t), c_2(t), ...\right]^\intercal$ con-

sistent with eq. (6).

We use a combination of second-order central differences, and first-order forward or backward differences to spatially discretise the derivative terms in eqs. (2) and (3). We discretise on a variable mesh with N internal nodes such that $n_i \approx n(\xi_i, \hat{t})$ and similar for c_i. The mesh is generated such that $\xi_{i+1} - \xi_i = 0.95(\xi_i - \xi_{i-1})$ and $\xi_1 = 10^{-9}$.

We use up-winding to approximate the derivative in the advection term in eq. (2) such that

$$\frac{\partial n_i}{\partial \xi} \approx \begin{cases} \dfrac{n_{i+1} - n_i}{\xi_{i+1} - \xi_i}, & \hat{v} - \xi \hat{R}'(t) < 0, \\ \dfrac{n_i - n_{i-1}}{\xi_i - \xi_{i-1}}, & \hat{v} - \xi \hat{R}'(t) > 0. \end{cases} \tag{7}$$

We approximate the remaining first and second derivative terms using a second-order central difference, such that

$$\frac{\partial^2 n_i}{\partial \xi^2} \approx \frac{\dfrac{n_{i+1} - n_i}{\xi_{i+1} - \xi_i} - \dfrac{n_i - n_{i-1}}{\xi_i - \xi_{i-1}}}{\dfrac{\xi_{i+1} + \xi_i}{2} - \dfrac{\xi_i + \xi_{i-1}}{2}}, \tag{8}$$

and

$$\frac{\partial n_i}{\partial \xi} \approx \frac{\dfrac{n_{i+1} + n_i}{2} - \dfrac{n_i + n_{i-1}}{2}}{\dfrac{\xi_{i+1} + \xi_i}{2} - \dfrac{\xi_i + \xi_{i-1}}{2}}. \tag{9}$$

At the left boundary, we approximate

$$n_0 \approx n_2 \text{ and } c_0 \approx c_2.$$

At the right boundary, we have that $c_{N+1} = 1$ and

$$n_{N+1} \approx n_N + 2(\xi_{i+1} - \xi_i) \frac{Q_p \hat{R}(t)}{D_p} (1 - p_0 - n_N).$$

2 Sensitivity matrix

Consider, for instance, $\mathbf{q}(\mathbf{p})$, from parameters in one model, $\mathbf{p}$, to parameters in another, $\mathbf{q}$. Then, from a first-order Taylor series approximation, we have that

$$\mathbf{q}(\mathbf{p}) \approx \mathbf{q}(\mathbf{p}_0) + J_{\mathbf{q}}(\mathbf{p}_0)(\mathbf{p} - \mathbf{p}_0). \tag{10}$$

Here, the rows of the Jacobian matrix correspond to gradients with respect to each component of $\mathbf{q}$. Without loss of generality, consider that $\mathbf{q}(\mathbf{p}) = [q(\mathbf{p}), ...]^\intercal$ where we have that

$$q(\mathbf{p}) \approx q(\mathbf{p}_0) + \nabla q(\mathbf{p}_0) \cdot (\mathbf{p} - \mathbf{p}_0). \tag{11}$$

We are interested in deriving an expression for *relative* changes with respect to $q(\mathbf{p}_0) = q_0$ and $\mathbf{p}_0$. Consider that

$$\frac{q(\mathbf{p}) - q_0}{q_0} = \frac{\nabla q(\mathbf{p}_0)}{q_0} \cdot (\mathbf{p} - \mathbf{p}_0).$$

Next, using the result that, for symmetric invertible matrix A, we have that $\mathbf{u}\cdot\mathbf{v} = (A\mathbf{u})\cdot(A^{-1}\mathbf{v})$,

$$\underbrace{\frac{q(\mathbf{p}) - q_0}{q_0}}_{r(\mathbf{q})} = \underbrace{\frac{D(\mathbf{p}_0)\nabla q(\mathbf{p}_0)}{q_0}}_{\mathbf{s}} \cdot \underbrace{\left[D^{-1}(\mathbf{p}_0)(\mathbf{p} - \mathbf{p}_0)\right]}_{\mathbf{r}(\mathbf{p})}.$$

We choose $D(\mathbf{p}_0) = \text{diag}(\mathbf{p}_0)$, so that $D^{-1}(\mathbf{p}_0)(\mathbf{p} - \mathbf{p}_0)$ is a vector composed of the relative difference between each component of $\mathbf{p}$ and $\mathbf{p}_0$. That is,

$$D^{-1}(\mathbf{p}_0)(\mathbf{p} - \mathbf{p}_0) = \mathbf{r}(\mathbf{p}) = \begin{bmatrix} \frac{p^{(1)} - p_0^{(1)}}{p_0^{(1)}} & \frac{p^{(2)} - p_0^{(2)}}{p_0^{(2)}} & \cdots \end{bmatrix}. \tag{12}$$

Therefore, approximate relative changes in the output, $r(q)$, are given by

$$r(q) = \mathbf{s} \cdot \mathbf{r}(\mathbf{p}). \tag{13}$$

From eq. (14) we can see that relative changes in $\mathbf{q}$ will be largest if $\mathbf{s}$ and $\mathbf{r}(\mathbf{p})$ are parallel, and smallest if $\mathbf{r}(\mathbf{p})$ and $\mathbf{r}(\mathbf{p})$ are orthogonal. If we let $\mathbf{r}(\mathbf{p}) = t\mathbf{s}$, then

$$r(q) = \mathbf{s} \cdot t\mathbf{s} = t\|\mathbf{s}\|^2 \Rightarrow t = \frac{r(q)}{\|\mathbf{s}\|^2}. \tag{14}$$

3 Sensitivity of the Greenspan to bounded Gompertz model-map

In the main text, we analyse the map between the Greenspan and logistic models to learn about non-identifiabilities in the Greenspan model. Here, we repeat this exercise using the bounded Gompertz model as a surrogate. The bounded Gompertz model fits with $\mathbf{p}_3 = \left[\lambda, R_{\mathrm{max}}\right]^\intercal = \left[1.00, 317\right]^\intercal$ and $R^2 = 0.99997$. The sensitivity matrix is given by

$$S_{13}(\mathbf{p}_1) = \begin{array}{c} \begin{matrix} \quad Q & \quad R_{\mathrm{d}} & \quad \gamma & \quad \lambda \end{matrix} \\ \begin{bmatrix} 0.0277 & 0.00256 & 0.0787 & 0.992 \\ 0.897 & 0.999 & -0.295 & 0.0143 \end{bmatrix} \end{array} \begin{matrix} \lambda \\ R_{\mathrm{max}} \end{matrix}, \tag{15}$$

and, similar to the map to the logistic model, shows that $(Q, R_{\mathrm{d}}, \gamma)$ relate to R_{max} and λ relates to λ. These relationships are clearer using the bounded Gompertz model compared to the logistic, particularly in relation to λ, since the crowding function for the bounded Gompertz and Greenspan models are more similar.

4 Sensitivity of the Greenspan to Richards model-map

In the main text, we analyse the map between the Greenspan and logistic models to learn about non-identifiabilities in the Greenspan model. Here, we repeat this exercise using the Richards model as a surrogate. The Richards model fits with $\mathbf{p}_4 = \left[\lambda, R_{\mathrm{max}}\beta\right]^\intercal = \left[1.11, 311, 1.28\right]^\intercal$ and $R^2 = 0.999$. The sensitivity matrix is given by

$$S_{14}(\mathbf{p}_1) = \begin{array}{c} \begin{matrix} \quad Q & \quad R_{\mathrm{d}} & \quad \gamma & \quad \lambda \end{matrix} \\ \begin{bmatrix} -0.121 & -0.0858 & -0.141 & 1.12 \\ 0.869 & 0.985 & -0.344 & 0.084 \\ 0.44 & 0.212 & 0.813 & -0.537 \end{bmatrix} \end{array} \begin{matrix} \lambda \\ R_{\mathrm{max}} \\ \beta \end{matrix}, \tag{16}$$

and, similar to the map to the logistic model, shows that $(Q, R_{\mathrm{d}}, \gamma)$ relate to R_{max} and λ relates to λ. We also see that nearly all parameters relate to the shape parameter, β.

References

[1] Ward JP, King JR. Mathematical modelling of avascular-tumour growth. II: Modelling growth saturation. IMA Journal of Mathematics Applied in Medicine and Biology. 1999;16(2):171–211.

[2] Rackauckas C, Nie Q. DifferentialEquations.jl – A performant and feature-rich ecosystem for solving differential equations in Julia. Journal of Open Research Software. 2016;5(1). doi:10.5334/jors.151.
