# Supplementary material for: Geometric analysis enables biological insight from complex non-identifiable models using simple surrogates
Source: PLoS Comput Biol. 2023 Jan 20;19(1):e1010844. doi: 10.1371/journal.pcbi.1010844 (PMC9891533; doi:10.1371/journal.pcbi.1010844)
Supplement: Attachment — Submitted filename: Responses.pdf [file pcbi.1010844.s002.pdf]

Referee 1 (Dr Ivo Siekmann)

In their manuscript “Geometric analysis enables biological insight from complex non-identifiable models using simple surrogates”, Browning and Simpson develop a new approach for investigating complex mechanistic models which are often non-identifiable by studying maps to simpler models with fewer parameters that are identifiable. I very much enjoyed reading this work - the approach seems to be technically reasonable, the manuscript is very well-written and the new method described here is highly original and looks to have quite a few promising applications.

I have some minor comments as well as a few general questions. These are mostly meant to be suggestions but it would be good if the authors could discuss these questions, even if only briefly.

Response: We thank Dr Siekmann for their positive review of our manuscript and now addressed their minor comments.

R1.1 Non-identifiability can indicate that some information about the system is irretrievably lost.

(a) The approach is based on the implicit assumption that at least some aspects of the processes represented in the more complex models can be inferred by fitting the model to the data available. I don't think that this is necessary true, even if we ignore non-trivial examples of models that are structurally non-identifiable. Looking at the example of a growing tumour spheroid investigated in this study it is a legitimate question to which extent the complex underlying processes of a growing tumour spheroid can possibly contained in highly simplistic experimental data only consisting of the tumour spheroid radius over time. For the Ward & King model this comes down to the assumption that we can gain essential information regarding the concentration profile of oxygen within the spheroid, just by observing how quickly the tumour expands.

Response: We agree that our approach does not increase the amount of information available in a given set of experimental data. However, our goals are aimed at understanding the effect model parameters, and the corresponding mechanisms, have on the model output and therefore macroscopic observations. For example, the parameters in the Ward and King model are largely non-identifiable, however it is useful to examine which parameters relate to the early-time growth rate, and which parameters relate to the largest spheroid size. In otherwords, we leverage mechanistic modelling to produce insights from data that would otherwise be lost if one were to go directly from a non-identifiable mechanistic model (such as the Ward and King model) to a one-dimensional metric (such as the likelihood). We now explicitly state this point in the revised manuscript (Page 26).

(b) The authors might argue that the question how much can be learnt about the parameters of a complex model from simple data is exactly what their approach addresses. But it is very likely that the dependency of a complex process such as the development of an oxygen gradient on the data will respond very sensitively if the model is changed. So another implicit assumption is that the complex model is a better or more accurate representation of the system than alternative models.

Response: Indeed, a challenge in mathematical biology is model specification and misspecification. As Dr Siekmann states, inferences can be entirely model-dependent, with parameter estimates themselves being entirely model dependent. However, it is nearly always the case that a number of models are proposed for a given set of data, and often these are of varying levels of complexity. Our approach aims to provide a method to interpret parameters in a complex, non-identifiable model that a modeller has decided appropriate for the system, using a simpler, identifiable model.

R1.2 (a) I disagree with the authors' presentation of the logistic model (and other related models) as purely phenomenological. The logistic model has a clear mechanistic interpretation of individuals competing for space.

Response: We agree that, in a more standard context, the logistic and related sigmoid growth models are mechanistic, and can be derived from individuals competing for space. However, we suggest that the logistic and related models are largely phenomenological in the context of tumour spheroid models, where cells grow as a single compact mass to a maximum spheroid size that isn't necessarily determined by the availability of space (e.g. nutrients rather than space could be the primarily limiting factor). We now explicitly state this context when presenting the logistic model in the revised manuscript.

(b) This matters because the authors state that the role of surrogate models like the logistic model is to identify "features" of the data. In the case of the spheroid data as far as I understand they refer to the growth rate and maximal radius. But the logistic model is more than a tool for inferring growth rate and maximal radius, it is also the result of a specific modelling decision of competition (in contrast to alternatives such as the whole class of Richards' models).

Response: We agree that, typically, the logistic model arises as a result of competition. However, in the context of spheroid models, growth ceases as a result of a variety of factors that cannot be captured by models that neglect space (oxygen and nutrient availability, mechanical factors, etc).

(c) In my opinion, it would be better to present the models in this paper as a hierarchy of mechanistic models - logistic and related models (Gompertz, Richards') explain the observed growth of tumour spheroid as the result of competition without explicitly representing its underlying cause (such as competition for nutrients or oxygen).

Response: In response to this comment, we state that the logistic and Gompertz models do not explicitly incorporate the biophysical mechanisms that influence growth, but rather describe behaviour using parameters such as the early-time growth rate and long-time limiting spheroid size (Page 6).

R1.3 The "complex" models in this papers are still quite simple and have relatively few parameters - the authors themselves mention some examples from the literature. It would be good if the authors could discuss if they expect that between-model Jacobians and sensitivities would still useful for much more complicated models (of course, these models would most likely have the issue mentioned in 1.).

Response: While we agree that the models considered have relatively few parameters, we disagree that the Ward and King model is simple in the context of typical spheroid

data. Given the impact that the Ward and King model has had on the mathematical community (evidenced by high numbers of citations and a high reputation within the spheroid modelling community), it is surprising to note that this model has never, to our knowledge, been used to interpret experimental data; likely driven, in part, by issues relating to parameter identifiability. Whether the Jacobians and sensitivity matrices are useful probably depends on how well the resultant model behaviour can be described by models with lower-dimensional parameter spaces (hence, our focus on the six models that are statistically indistinguishable from commonly reported experimental data).

R1.4 Minor comments (part 1):
Note that for very minor comments, we do not provide a page or margin reference.

(a) p.3, above equation (1): ”In practise” $\rightarrow$ maybe ”In practice”?

Response: We have addressed this in the revised manuscript (Page 3).

(b) p.3, last paragraph: ”We generate synthetic radius measurements... to the complex multiphase model of Ward and King [15,16]” - repeated in similar words on page 6.

Response: To avoid repetition, we have revised the wording of the sentence on Page 6.

(c) p.5, ”local-curvature” $\rightarrow$ maybe ”local curvature”?

Response: We have addressed this in the revised manuscript (Page 5).

(d) p.5, ”Geometrically, the FIM is related to the Hessian of the log- likelihood function and so contains information about the directions in parameter space in which the log-likelihood (and therefore the model) is both sensitive and insensitive [26]” - this sentence is unclear, what does ”both sensitive and insensitive” mean? (although it becomes somewhat clearer after reading the next sentence)

Response: We have reworded this sentence in the revised manuscript (Page 5).

(e) p.6, Figure 2b: The figure shows a map from a non-identifiable model 3 to another non-identifiable model 2 which is then mapped to an identifiable model 1. As far as I understand in this manuscript the authors have only studied maps of one non-identifiable model to another identifiable model. Would it make sense to extend this process by constructing maps between non-identifiable models?

Response: While we suggest that, given we consider both the radial-death and logistic model in sequence to interpret the non-identifiable Greenspan model, and then interpret the radial-death model with the logistic model, we do study a map between more than two models. However, we agree that it is misleading to have an ambiguous ”model 2” in Figure 2 that could be identifiable or non-identifiable, and so have simplified the figure accordingly (Page 6).

(f) p.6, third line from the bottom: ”Aside from the requirement that models are deterministic...” - Is this constraint necessary? I do actually think that this approach could be extended to stochastic models.

Response: While it may be possible to apply our approach to stochastic models, it does not readily generalise as we require that model-map Jacobians be available (although, one could consider the *expected value* of a stochastic model, which in itself could be thought of as a deterministic model) We now state the explicit requirement in the revised manuscript (Page 7).

(g) p.7: "The choice of $R_0 = 10\,\mu\text{m}$ is made to ensure that the simple models we consider are identifiable." - It is not clear to me how identifiability depends on the initial condition.

Response: We have clarified that we set $R_0 = 10\,\mu\text{m} \ll R_{\text{max}}$, which ensures parameters in simpler models (i.e., the proliferation rate in the logistic model) are identifiable (Page 7). If this were not the case, parameters that play roles in primarily the early-time dynamics may not be identifiable.

(h) p.7, last few sentences above "Model 1." - I think that the authors might consider simplifying this paragraph. I think it would be clearer to state that time and (if applicable space) are deliberately not non-dimensionalised.

Response: We have simplified these two sentences in the revised manuscript (Page 7).

(i) p.9: "The logistic model and its generalisations (including the Gompertz and Richards models [42]) are purely phenomenological; they are not explicitly constructed from biological mechanisms by which overall growth is inhibited and eventually ceases." - as mentioned above in general comment 2. I don't think that this is true, it is possible to derive logistic growth as the result of competition for space.

Response: We have clarified that these models are purely phenomenological *in the context of tumour spheroid models*, and we agree with the referee that in many contexts, such models can be derived from a competition for space (Page 9).

(j) p.9: "While logistic growth is commonly used to describe the time-evolution of spheroid volume [11], we find that logistic growth in the spheroid radius is more consistent with our synthetic data": I am not sure if I fully understand this sentence. Do the authors want to say that the logistic growth model is more commonly used for modelling tumour volume rather than radius?

Response: Typically, the logistic growth model is used to describe the time-evolution of *spheroid volume*. However, such models are largely phenomenological in the context of spheroid modelling and we find that that the *spheroid radius* is better described by the logistic growth model (Fig 1, Page 4). We have rephrased this sentence in the revised manuscript (Page 10).

(k) p.11: "However, the logistic model differs in that it does not include a discrete initial phase where growth is exponential." - I am not sure if "discrete" is the right word here? I assume that this is in comparison with the bounded Gompertz model where the radius grows exponentially for $0 < R(t) < R_{\text{max}}/\exp(1)$?

Response: We have rephrased this sentence to clarify that the logistic model "does not include an initial period of time where growth is exponential (Page 11).

(l) p.12, below eq. (12): Although equation (14) can, of course, be derived without explicitly stating the rate g of cells entering the necrotic core it would be good to state some characteristics, for example, that $g(V_1) = 0$ if $R(t)$

Response: We sincerely thank Dr Siekmann for bringing this omission to our attention, and have clarified that $g(V_1) = 0$ for $R(t) < R_\mathrm{d}$ in the revised manuscript (Page 12).

(m) p.12, eq. (14): Also, in eq. (14) I assume that the terms involving $R(t) - R_d$ vanish for $R(t)$

Response: As with our response to R1.4l, we have clarified this in the revised manuscript (Page 12).

(n) p.13, below eq. (16): I find the description of the Ward & King model a bit terse. Without knowing the model the role of the "cellular material" is unclear, and, similarly, what it means that it is "assumed to have the same density as living cells". Also, for readers not familiar with moving boundaries, eq. (17) might be hard to understand without a bit more explanation.

Response: We now clearly state what we mean by "cellular material" in the revised manuscript (Page 12). Further, we provide brief text to help the reader understand the system of equations associated with the Ward and King model, specifically, how the moving boundary problem relates to the overall growth of the spheroid differently to other models (Page 13).

(o) p.14: under "Profile likelihood": "constrain" $\rightarrow$ constraint

Response: We have addressed this in the revised manuscript (Page 15).

(p) p.14, eq. (22): It would be helpful to remind the readers that $\hat{p}_i$ is the maximum-likelihood estimator when all parameters are varied.

Response: We have addressed this in the revised manuscript (Page 15).

(q) p.15, top: "...comparing the profile log-likelihood of a parameter to the threshold for an approximate 95% confidence interval,..." Would it be simpler to say that identifiability is defined based on constructing approximate confidence intervals for each parameter?

Response: As we never explicitly calculate 95% confidence intervals for each parameter (and rather rely on direct comparison with the profile likelihood), we believe that the current text is the best explanation for our approach.

(r) p.15, "We produce points on the profile likelihood sequentially, using the log-likelihood at the MLE, the previous point, and the initially specified guess as an initial guess; ..." This is very unclear.

Response: We have rephrased this sentence in the revised manuscript (Page 15).

(s) p.15, bottom and p.16, caption of Figure 5: It is not clear to me what ”profiled error function” means. Also, it would be good to mention in the Figure caption that Figure 5e shows the noise-free case, in contrast to the other panels.

Response: We have revised the text in the manuscript to make this clear (Page 17). We also now state in the caption for Fig 5 that profiles of the error function are produced using noise-free data (Fig 5, Page 16).

(t) p.19: ”Furthermore, this analysis demonstrates that, roughly speaking, the parameter subset (Q, R_d, γ) correspond primarily to the maximum spheroid size. In other words, there exists a trivariate function of (Q, R_d, γ) that maps to R_{max}, and by extension the likelihood.” - It is not clear to me how the authors come to this conclusion, here, or in similar cases below. I assume that it is related to implicit assumptions which components of the sensitivity matrix are ”small” - I think this should be explained explicitly.

Response: We now explicitly state how this conclusion is drawn in the revised manuscript (Page 19).

(u) p.19, ”... a direction in which the model relatively insensitive” $\rightarrow$ a direction in which the model IS relatively insensitive

Response: We have addressed this in the revised manuscript (Page 20).

(v) p.21, second line below subsection heading: ”with respect to model complexity and identifiable” $\rightarrow$ with respect to model complexity and IDENTIFIABILITY

Response: We have addressed this in the revised manuscript (Page 22).

(w) p.22, above subsection heading: ”In this case, we solve eq. (32) using the FIM for the radial-death model, and terminate the solution where the solution drops below the profile- likelihood-based threshold for a 95% confidence interval.” - I have no idea what the authors want to say here.

Response: We have rephrased this sentence in the revised manuscript (Page 22). In effect, we show the resultant curve within a 95% likelihood-based confidence region in the three-dimensional parameter space (outside this 95% confidence region, points on the curve are inconsistent with the data).

(x) p.23, ”The reason for number of parameters in the Ward and King model is the biological granularity it provides:” - This sentence is unclear and might be incomplete. Do the authors explain why the Ward and King model has more parameters than other models investigated here?

Response: We have replace the word “granularity” with the phrase “number of biological mechanisms” to make our meaning behind this point clear (Page 24).

(y) p.25: ”... we can use the rows sensitivity matrix, ...” - incomplete sentence

Response: We have addressed this in the revised manuscript (Page 25).

(z) p.25: ”... , mathematical models can always be made arbitrarily complex.” - I think that GOOD modellers would not make models arbitrarily complex.

Response: We agree with Dr Siekmann, however, our point is that mathematical models can almost always be made more complex than might be suitable for even highly detailed experimental data. This can even be inadvertent: as stated in our response to R1.3, the Ward and King model has relatively few parameters, however has not yet, to our knowledge, been used to quantitatively interpret experimental data.

R1.5 Minor comments (part 2):
Note that for very minor comments, we do not provide a page or margin reference.

(a) p.25, ”... nutrient dependancies...” → nutrient dependencies

Response: We have addressed this in the revised manuscript (Page 26).

(b) p.25, bottom line: ”... however not being constructionist, ...” - Maybe ”constructivist”?

Response: We agree that “constructivist” is perhaps clearer (Page 26).

(c) p.25-26, bottom to top: ”... they limit the conversation between the mathematics and the biology that allows impactful insights to be made.” - This sentence sounds a bit bombastic :-)

Response: We have rephrased this sentence in the revised manuscript (Page 26).

(d) p.26: ”... objectively studying the geometry of a map from the parameters in the complex model to those in the identifiable surrogate.” - What does ”objectively” mean in this case?

Response: We agree, the word “objectively” isn’t needed in this sentence, and has been removed from the revised manuscript (Page 26).

(e) p.26: ”One interpretation of our approach is of a layer that sits between the model parameters and the likelihood (or other goodness-of-fit metric) that is traditionally studied in identifiability and model sloppiness analysis.” - I am not sure what the authors want to say here.

Response: We have rephrased this sentence in the revised manuscript to state that one can thing of our approach as a way to “provide an intermediate mode of interpretation that sits between the model parameters and the likelihood” (Page 26).

(f) p.26: ”... data from these experiments can be even more limited” - What do the authors want to say here? Does this mean that these data sets are rare?

Response: We have rephrased this sentence to state that data are “even more limited, noisy, or sparse in comparison with experimental models of avascular tumour growth” (Page 27).

(g) p.26: ”..., if the complex model is considered reality, the surrogate model produces predictions that are biased” - I am not sure what this means. Which parameters in the surrogate model are biased and why?

Response: In most cases the surrogate model is not equivalent to the complex model in the noise-free limit: i.e., if the complex model produces “reality”, then this “reality” cannot be reproduced *exactly* by the surrogate model and so predictions made from the surrogate model will be biased.

(h) p.26: ”... implicitly incorporates the noisy nature of the experimental measurements.” - what does this mean?

Response: We have removed this sentence from the revised manuscript to avoid confusion and make the discussion in this paragraph clear (Page 27).

(i) p.27: ”Recent work considers identifiability and sloppiness analysis based directly on the parameter covariance matrix estimated from Bayesian methods such as Markov-chain Monte-Carlo to provide an overall snapshot of the global parameter sensitivities. However, we expect this approach to be problematic in our geometric framework, since the model-map is based on an equivalence between models that may only apply locally.” - I find it hard to follow this argument. Why do the authors think that Bayesian approaches might not be applicable and what do they mean when they say that equivalence between models ”may only apply locally”?

Response: It is the “apply locally” constraint that concerns us most with utilising the Bayesian approach that uses the covariance matrix instead of the FIM. This is because the model-map Jacobian typically would only apply in the region of the parameter set used to generate it. We now state this clearly in the revised manuscript (Page 27).

(j) p.28: ”Building up a global model-map between the complex and surrogate models is another approach to overcome the localisation limitation of our methods, and could be computationally advantageous in the case of computationally expensive complex models.” - What does ”localisation limitation” mean?

Response: We refer to our response to R1.5i, regarding a clarification of what we mean by “localisation limitation”.

(k) p.28: ”...complex models are numerous and conformable to the questions of interest” - What does ”conformable to the question of interest” mean?

Response: We have rephrased this sentence in the revised manuscript to make its intended meaning clearer (Page 28).

Referee 2

Authors also provide compelling explanation of the limited utility of AIC when fitting mechanistic models to data (e.g. models with high AIC values may provide some utility if parameters of interest are identifiable). The authors then propose a method to study models with non-identifiable parameters using identifiable models w/ similar behavior. The models introduced in figure 1 have indistinguishable AIC, and model equivalence can be studied. The authors

approach to begin with a model of intermediate complexity (Greenspan) then add noise and fit alternative models to this synthetic data, is a nice approach to understand the utility of their methods, with some objective ground truth known. The papers exposition is generally very clear and the results and methods are exciting & a good fit for publication at PLOS CB. For example, the comparison between models (e.g. eqn 31) is a powerful and intuitive result from the methods proposed here.

Below I outline a few minor suggests to improve the clarity of the manuscript. These are all minor corrections to help clarify a few points that were initially confusing but clarified in later points of the manuscript.

Response: We thank the referee for their positive report, and we now address their specific comments.

R2.1 Equation 2 implies that this method is used to fit the model to one output data only. Can this method be extended to multiple data streams collected simultaneously (e.g. tumor radius and nutrient level simulataneously)? I think some brief discussion of this should be mentioned in the intro and/or discussion.

Response: The link between the model and data output, defined by the function $\mathbf{m}_i(\mathbf{p})$ can be extended readily to include multiple data streams. For example, in Fig 6 (Page 21C) we include both outer radius measurements and the final necrotic core size. We now state this possibility at the introduction of Equation 2 to make this point clear (Page 7).

R2.2 Page 7, "...which is consistent with experiments where these quantities are not directly measured." Perhaps its just the wording of this sentence, but I am confused about this sentence. Do the authors mean that every dependent variable that does not have a corresponding experimental value for comparison is non-dimensionalized?

Response: In response to R1.4h we have rephrased the sentences at the end of this paragraph to make the meaning clear (Page 7)

R2.3 Page 7, the phrase "which we have previously validated against experimental data" is repeated twice (first & fourth paragraphs) - probably only necessary to state once.

Response: We now state this phrase only once in the revised manuscript.

R2.4 Page 14, second last paragraph: "...the MLE subject to the constrain" I think this should be "constraint" not "constrain".

Response: We have corrected this typographical error in the revised manuscript (Page 15)

R2.5 Section 3.1.1 can be improved by clarifying the description of identifiability in figure 5. For example "we can establish identifiability by comparing the profile log-likelihood of a parameter to the threshold for an approximate 95% confidence interval" it was not

initially clear to me how the "comparing the profile" was done. Are we looking for ranges where PLL $>$ 95% or PLL $<$ 95%? Figure 5 is a great visual, supporting eqn. 22, but more brief explanation would help the reader. I also think it would be helpful to briefly note how identifiability would be altered w/ a new confidence interval (perhaps in the caption of figure 5).

Response: We make the process of comparison clear in the revised manuscript (Page 15). Specifically, we classify parameters as identifiable if we observe a tightly-constrained interval for which the profile likelihood is above the threshold. We also comment on how this threshold might change for different levels of confidence interval in the caption of Fig 5 (fig. 5).

R2.6 After reading subsequent sections, I think the text on page 5, 1st paragraph, last sentence can be clarified: "... relatively large eigenvalues (informative directions) separated by several decades from one or two sloppy directions." It wasnt initially clear to me if 1) the separation or 2) the magnitude is the indicator of informative vs non-informative (sloppy) directions. Im still unclear how this related to the claim on page 19 that the "dimensionality of this manifold corresponds to the number of sloppy directions" with reference to figure 1d. From visual inspection of Figure 6, Im not sure if I'm drawing the correction conclusion about the dimensionality of the manifold (as each subpanel appears to be a one-dimensional manifold).

Response: We clarify these points in the revised manuscript (Page 5). Indeed, it is difficult to interpret the separation of eigenvalue magnitudes for models with only two parameters (i.e., the logistic model used in the Fig 5 example) since the relative difference can be changed arbitrarily through parameter rescaling. It is much easier to interpret results for, i.e., the Greenspan model, where results in Fig 1D show two groupings of eigenvalues (three that are relatively large, and only one that is small). The single small eigenvalue indicates a single "sloppy" direction or one-dimensional manifold in which the likelihood changes very slowly.

Referee 3 (Dr Alejandro Villaverde)

This is an interesting and intriguing paper. Its main contribution is a method to map parameters (or combinations thereof) between different models. Thus, some kind of correspondence between the parameters in a complex, unidentifiable model and the parameters in a simple identifiable model can be established. This is useful because sometimes a parameter appearing in two different models under the same name is intuitively / conceptually thought to play the same role in both models (i.e. to have the exact same physical meaning), but this is not necessarily so. Importantly, the simpler (also called surrogate) model does not need to be nested within the complex model.

Response: We thank Dr Villaverde for their encouraging and helpful review, and we now address their specific comments.

R3.1 My main concern is about the organization of the article:

(a) While the paper is, generally speaking, well written, I found it too lengthy. I think it is difficult to grasp the precise nature of the main contribution, and it is easy to lose

track of the main message during the reading. Perhaps the authors could consider moving some material to the Supplementary Information file. For example, Section 2.1 (Models) could be shortened by doing so, leaving only a very brief description of the models (maybe a Table would be useful) as well as the figures and tables.

Response: We agree that the models section is lengthy, however we are of the opinion that these models may not be familiar to many readers of *PLOS Computational Biology*, and therefore believe that the paper is clear leaving full model details in the main text.

(b) It is a bit weird that Section 2, "Methods", has only one subsection, which describes models but not methods. Instead, identifiability methods are described in Section 3.1. I would suggest moving the description of those methods to a new subsection 2.2, and leaving 3.1 to the application of the methods to the models.

Response: We agree that it is strange that Section 2 contained only the one subsection "Mathematical Models". Upon reviewing the formatting requirements for *PLOS Computational Biology*, we have renamed Section 2 "Models", and deleted the subsection title.

(c) As for Subsection 3.2, it presents the methodology with which the model-parameter map is obtained, which is the main contribution of the article. I think this should be highlighted more. Also, the methodology should be clearly defined, in the most general way possible, and with sufficient details. If I understood it correctly, the main result would be the construction of the sensitivity matrix (eqs. 2930), which is obtained from the Jacobian in eq. (28). The calculation of said Jacobian is not trivial at all, and more details should be given about it.

Response: Indeed, from the current organisation of the Results section it is unclear whether the novelty of the techniques lie. We have rephrased the beginning of this section to clearly divide Results into two subsections: first, a standard analysis using existing techniques, and secondly, the novel geometric analysis that is developed in our work (Page 14). We believe that the current organisation is important, as the standard approaches (some of which may be unfamiliar to many readers of *PLOS Computational Biology*) provide a motivation and contrast for the geometric approach that we develop.

Regarding the calculation of the Jacobian, we use an existing software package (`FiniteDifferences.jl`) that is robust to numerical noise introduced by the optimisation algorithm (Page 19). We take great care to validate the calculated Jacobian graphically (in, i.e., Fig 6, Page 21) and numerically (by, i.e., examining the relationship between the model-map Jacobian and the eigenvectors of the FIM in Table 2, Page 25).

R3.2 I also have a number of more specific comments:

(a) Page 1: I am not fully convinced by this part of the last sentence in the Abstract: "Our geometric approach is able to (...) subset non-identifiable parameter combinations that relate to individual data features (...)". It is unclear to me how parameter combinations relate to "individual data features". If this sentence is kept, the authors should better explain it in the article.

Response: We have expanded this sentence in the revised abstract to clarify that by "individual data features" we mean features in the data characterised by parame-

ters in a surrogate model; the maximum size of the spheroid, the early-time growth rate, for example (Page 1).

(b) Page 4: "In comparison to model selection criterion like AIC, identifiability analysis provides an often subjective view of the identifiability of individual model parameters." I find it a bit weird to use the word "subjective" here.

Response: We agree, our meaning with the word "subjective" is unclear, and have reworded this sentence in the revised manuscript to provide a more clear contrast between identifiability analysis (that provides information about individual model parameters) and model selection criterion (that look at models as a whole) (Page 4).

(c) Page 9, equation (4): is Rn(t) = 0? If yes, then subsequent equations would be simplified...

Response: We thank the Dr Villaverde for bringing this omission to our attention, and refer them to comments R1.4l and R1.4m above.

(d) Page 19: in the paragraph immediately before section 3.2.1 it is argued that the approach can be used to finding identifiable combinations of individually unidentifiable parameters. (Thanks to the map from a simple, identifiable model to a complex, unidentifiable one). The authors could mention that there are already other methods to achieve that goal, e.g. based on Profile Likelihood (`https://doi.org/10.1371/journal.pone.0162366`) or FIM-based sensitivity analysis (see `https://doi.org/10.1016/j.automatica.2015.05.004` and more recent works by the first author).

Response: We thank Dr Villaverde for bringing these papers to our attention and now cite them appropriately alongside the reference to Marisa Eisenberg's subset profiling paper in the revised manuscript (Page 19).

(e) Page 19: "Furthermore, this analysis demonstrates that, roughly speaking, the parameter subset (Q; Rd; gamma) correspond primarily to the maximum spheroid size." Why is it so? It is not obvious to me.

Response: We refer Dr Villaverde to our response to comment R1.4t above. In summary, it is based upon the magnitude of the relevant elements in the model-map sensitivity matrix.

(f) Page 21: "Secondly, we see that gamma and lambda in the Greenspan model have a near one-to-one correspondence to zeta in the radial-death model." This does not seem right; shouldnt it be only gamma, not gamma and lambda?

Response: We thank Dr Villaverde for raising this point. These results highlight that parameter estimates are often model specific. In particular, in the Greenspan model the equivalent parameter ζ is related to the others by $\zeta = \lambda\gamma$, and so increasing either λ or γ will result in an increase to ζ. We have added ζ to the model parameter table, from which it was originally erroneously excluded, to make this clear (Page 10).

(g) Page 22: “... directions (if any) in the parameter space to which the model is insensitive...” it should probably be specified that what is insensitive is “the model output”, not simply “the model”.

Response: We have addressed this comment in the revised manuscript (Pages 5, 5, and 22).

(h) Page 27: “Recent work considers identifiability and sloppiness analysis based directly on the parameter covariance matrix estimated from Bayesian methods...”. Please provide references to that work.

Response: This was an unintentional omission in the original manuscript. We now provide the relevant citation in the revised manuscript (Page 27).

(i) A few typos:

- In page 3, “One of many criterion used...” should be “criteria”.
- In page 17, “For example, find that the condition number...” should be “For example, we find...”, or something similar.

Response: We have addressed these comments in the revised manuscript, and have thoroughly proofread the text.
